# Interarm blood pressure difference and risk assessment: the ECHORN cohort study

**DOI:** 10.3399/BJGPO.2024.0246

**Published:** 2025-12-19

**Authors:** O Peter Adams, Deron Galusha, Josefa L Martinez-Brockman, Euclid H Morris, Rohan G Maharaj, Cruz M Nazario, Maxine Nunez, Marcella Nunez-Smith

**Affiliations:** 1 Faculty of Medical Sciences, The University of the West Indies, Cave Hill Campus, Cave Hill, Barbados; 2 Five Islands Campus, The University of the West Indies, Five Islands Village, Antigua and Barbuda; 3 Equity Research and Innovation Center,Yale School of Medicine, New Haven, United States; 4 Department of Internal Medicine,Yale School of Medicine, New Haven, United States of America; 5 Department of Paraclinical Sciences, University of the West Indies, Saint Augustine Campus, Saint Augustine, Republic of Trinidad and Tobago; 6 Department of Biostatistics and Epidemiology,Graduate School of Public Health, University of Puerto Rico at Medical Sciences Campus, San Juan, Puerto Rico; 7 School of Nursing, University of the Virgin Islands, St. Thomas, United States

**Keywords:** clinical (general), diagnosis, blood pressure, hypertension

## Abstract

**Background:**

Guidelines recommend measuring blood pressure (BP) in both arms and using the higher reading.

**Aim:**

To determine interarm pressure difference (IAD) distribution and associated factors, and BP and atherosclerotic cardiovascular disease (ASCVD) risk classification using the higher and lower readings.

**Design & setting:**

This cohort study used a representative cross-sectional sample of community-dwelling residents aged ≥40 years on four Caribbean islands (Barbados, Puerto Rico, US Virgin Islands, and Trinidad).

**Method:**

BP was measured simultaneously in both arms. Mixed effects logistic and linear regression tested associations with an IAD. BP and ASCVD risk were classified using the higher and lower BP.

**Results:**

Of 2912 participants (mean age 57.2 years), 10.7% (95% confidence interval [CI] = 9.6 to 11.8) and 3.3% (95% CI = 2.6 to 3.9) had systolic IADs ≥10 mmHg and ≥15 mmHg, respectively, and 5.0% (95% CI = 4.2 to 5.8) and 1.8% (95% CI = 1.3 to 2.3) diastolic IADs ≥10 mmHg and ≥15 mmHg, respectively. Independent associations with systolic and diastolic IADs ≥10 mmHg and/or continuous outcomes, included increasing body mass index (BMI), systolic and diastolic pressures and hypertension (*P*<0.05). Higher versus lower arm BP reclassified 10.3% (95% CI = 7.8 to 12.8) and 8.3% (95% CI = 5.9 to 10.7) from below to above the 130-mmHg and 140-mmHg systolic thresholds, respectively, 10.8% (95% CI = 8.2 to 13.3) and 6.9% (95% CI = 4.9 to 8.9) at the 80-mmHg and 90-mmHg diastolic thresholds, respectively, and 9.2% (95% CI = 0.0 to 18.4) of those with an IAD of ≥10 mmHg at the ≥10% 10-year ASCVD risk threshold.

**Conclusion:**

Assessing both arms detects an IAD ≥10 mmHg and reclassifies BP in about 1 in 10 people. Increasing BMI and BP increase the risk of an IAD ≥10 mmHg or 15 mmHg.

## How this fits in

Guidelines recommend measuring blood pressure (BP) in both arms initially and using the higher reading arm subsequently, but few physicians do this. BP is variable but much of the data comes from sequentially measured systolic BP, does not estimate atherosclerotic cardiovascular disease (ASCVD) risk for the subset with a significant interarm BP difference (IAD), and does not include people from the Caribbean. Our study addressed these issues in a community-dwelling Caribbean population aged ≥40 years. We found ≥10 mmHg systolic and diastolic IADs in 10.7% and 5.0% of our sample, respectively, reclassified diagnosis and control status at the 140 mmHg systolic and 90 mmHg diastolic threshold in 8.3% and 6.9%, respectively, using the higher versus lower reading arm BP, and for those with a interarm systolic difference of ≥10 mmHg the higher versus lower BP moved an additional 9.2% above the 10% 10-year ASCVD risk threshold.

## Introduction

Hypertension practice guidelines state that blood pressure (BP) should be measured in both arms initially and the higher reading arm used subsequently.^1–5^ Few primary care physicians do this.^6,7^ A review of US,^1^ European,^2^ British,^3^ Australian,^4^ and World Health Organization (WHO) guidelines^5^ reveals that the recommendations have some differences. A minimum interarm BP difference (IAD) that makes subsequent use of the higher reading arm necessary is not indicated by some guidelines^1,2,5^ but others specify a difference of >5 mmHg^4^ or >15 mmHg.^3^ Two guidelines state that systolic differences >15 mmHg are associated with significant cardiovascular risk.^2,3^ One guideline indicates that while preferable, it may not always be practical to measure both arms initially.^5^ Another guideline indicates the IAD stipulation applies to systolic readings and should be established by simultaneous measurement.^2^ BP is variable making IADs based on sequential measurement less reliable than simultaneous measurement and may overestimate IADs.^8–10^

Using the higher versus lower arm systolic reading will move 12.4% and 11.9% of people, respectively, over the 130 mmHg and 140 mmHg systolic hypertension diagnosis or control thresholds, and 3.4% from below to above the 10% 10-year ASCVD risk threshold.^11^ A 10 mmHg systolic IAD has been proposed as the upper limit of normal.^12^ Several studies evaluate this threshold. It predicts both increased cardiovascular^12,13^ and all-cause mortality.^11,12^ Increasing age,^14–16^ diabetes, hypertension,^15–17^ increasing systolic BP,^15^ increasing total cholesterol, obesity or increasing body mass index (BMI),^15,17^ and decreased ankle brachial pressure index^17^ are associated with systolic IADs of ≥10 mmHg.

The Eastern Caribbean Health Outcomes Research Network (ECHORN) cohort study recruited adults on the Caribbean islands of Barbados, Puerto Rico, US Virgin Islands, and Trinidad. Between 2013 and 2018, 2961 persons were recruited to wave 1 of the cohort. This cohort has a high prevalence of hypertension and diabetes, and a peripheral arterial disease prevalence of 4.4%.^18,19^ Since previous studies have not been done in the Caribbean and because the choice of arm may impact patient management decisions, we aimed to determine the (a) proportion of the cohort with ≥5 mmHg, ≥10 mmHg, ≥15 mmHg, and ≥20 mmHg systolic and diastolic IADs, (b) factors independently associated with IADs of ≥10 mmHg and ≥15 mmHg, and (c) impact of using higher versus lower arm readings on the proportions of the cohort above established hypertension diagnostic and control, and ASCVD risk thresholds.

## Method

Stratified multi-stage probability sampling of community-dwelling residents aged ≥40 years was used on the islands of Barbados, Puerto Rico, and Trinidad and simple random sampling on US Virgin Islands. After obtaining informed consent, a survey and clinical examination were completed and blood tests done. Full details are reported elsewhere.^18^ BP was measured simultaneously in both arms using a Microlife WatchBP office automated oscillometric sphygmomanometer after participants had sat quietly for 5 minutes with their back supported, feet on the ground, legs uncrossed, and cuffs at heart level. Three readings were done automatically 1 minute apart, and for each arm the average was used.

Education level was classified as not completing high school, completing high school, some college, which could include having an associate degree, and having a university degree. Heart disease was defined as a self-report of one or more of the following conditions: coronary heart disease; angina pectoris; abnormal heart rhythm; heart attack; or congestive heart failure. BMI categories were normal or underweight <25 kg/m^2^, overweight 25–29.9 kg/m^2^, and obese ≥30 kg/m^2^.^2,20^ Hypertension was defined as a BP ≥140/90 mmHg in the arm with the higher systolic pressure and/or self-reported antihypertensive medication use. Diabetes mellitus was defined as one of the following: self-report of a healthcare worker diagnosis or diabetes medication use; an HbA1*c* ≥6.5% or fasting glucose ≥7.0 mmol/l. An elevated waist-to-hip-ratio was defined as >0.9 for men and >0.85 for women.^21^ Physical activity was estimated by the WHO Global Physical Activity Questionnaire.^22^ ASCVD risk was calculated according to American College of Cardiology/American Hypertension Association (ACC/AHA) guidelines.^23^

### Analysis

Data were analysed using SAS (version 9.4). The prevalence and 95% confidence interval (CI) of systolic and diastolic IADs of ≥5 mmHg, ≥10 mmHg, ≥15 mmHg, and ≥20 mmHg were determined. Potentially predictive demographic, medical history, anthropometric, and laboratory factors were compared between those with and without systolic and diastolic IADs ≥10 mmHg and ≥15 mmHg, and *P*-values calculated.

Mixed effects logistic regression was done with island as a random effect and other potential independent variables added as fixed effects. Associations involving a systolic IAD of ≥10 mmHg with a *P*-value <0.2 were used to create, through backward elimination, final models containing only variables with *P*-values <0.05. Hypertension, systolic and diastolic blood pressure are correlated and were therefore put into separate models. BMI was entered as a continuous variable. A similar analysis was repeated for diastolic IADs ≥10 mmHg and systolic and diastolic IADs ≥15 mmHg as the dependent variables.

Dichotomisation of IAD into thresholds aligns with hypertension guidelines and previous studies. However, it reduces variability in data and impacts statistical power.^24^ We therefore also conducted mixed effects linear regression of systolic and diastolic IAD as continuous dependent variables. For each model the Akaike information criterion (AIC; higher number indicates better model fit) and intraclass correlation coefficient (proportion of the variance owing to the random effect variable) were calculated.

Using the higher and lower systolic readings of each participant, the proportion (95% CI) of participants at or above the 130 mmHg and 140 mmHg systolic, and 80 mmHg and 90 mmHg diastolic thresholds were determined. For each threshold, the difference in the proportions (95% CI) between the higher and lower reading arms were calculated. Guidelines recommend management changes based on these thresholds.^1–5^

Ten-year ASCVD risk from ≥7.5% to <20% is considered intermediate by ACC/AHA cardiovascular disease prevention guidelines^25^ and their BP guidelines recommend possible treatment changes with ≥10% risk.^1^ The proportions (95% CI) of the sample with 10-year ASCVD risks ≥7.5% and ≥10% were calculated using the higher and lower systolic readings and differences in proportions (95% CI) between arms determined. The same comparison was repeated only for those participants with 10 mmHg systolic IADs.

## Results

Of 2961 cohort members, 2912 (mean age 57.2 years, 65.0% female, mean BMI 29.2 kg/m^2^, 41.3% on antihypertensive medication, and 15.6% reporting a heart condition) had valid systolic BP measurements and 2910 valid diastolic measurements (Supplementary table 1).

### Right versus left arm BP

Mean right and left arm systolic pressures were 135.3 (standard deviation [SD] 21.3) and 135.4 (SD 21.4) mmHg, respectively (*P* = 0.463). Mean right and left arm diastolic pressures were 80.4 (SD 11.6) and 80.3 (SD 11.4) mmHg, respectively (*P* = 0.284).

### Interarm BP differences (higher versus lower reading arm)

The difference between the mean higher and lower systolic and diastolic BP readings were 4.5 (137.6 versus 133.1) (*P*<0.001) and 3.1 (81.9 versus 78.8) mmHg (*P*<0.001), respectively.

**Figure 1.**
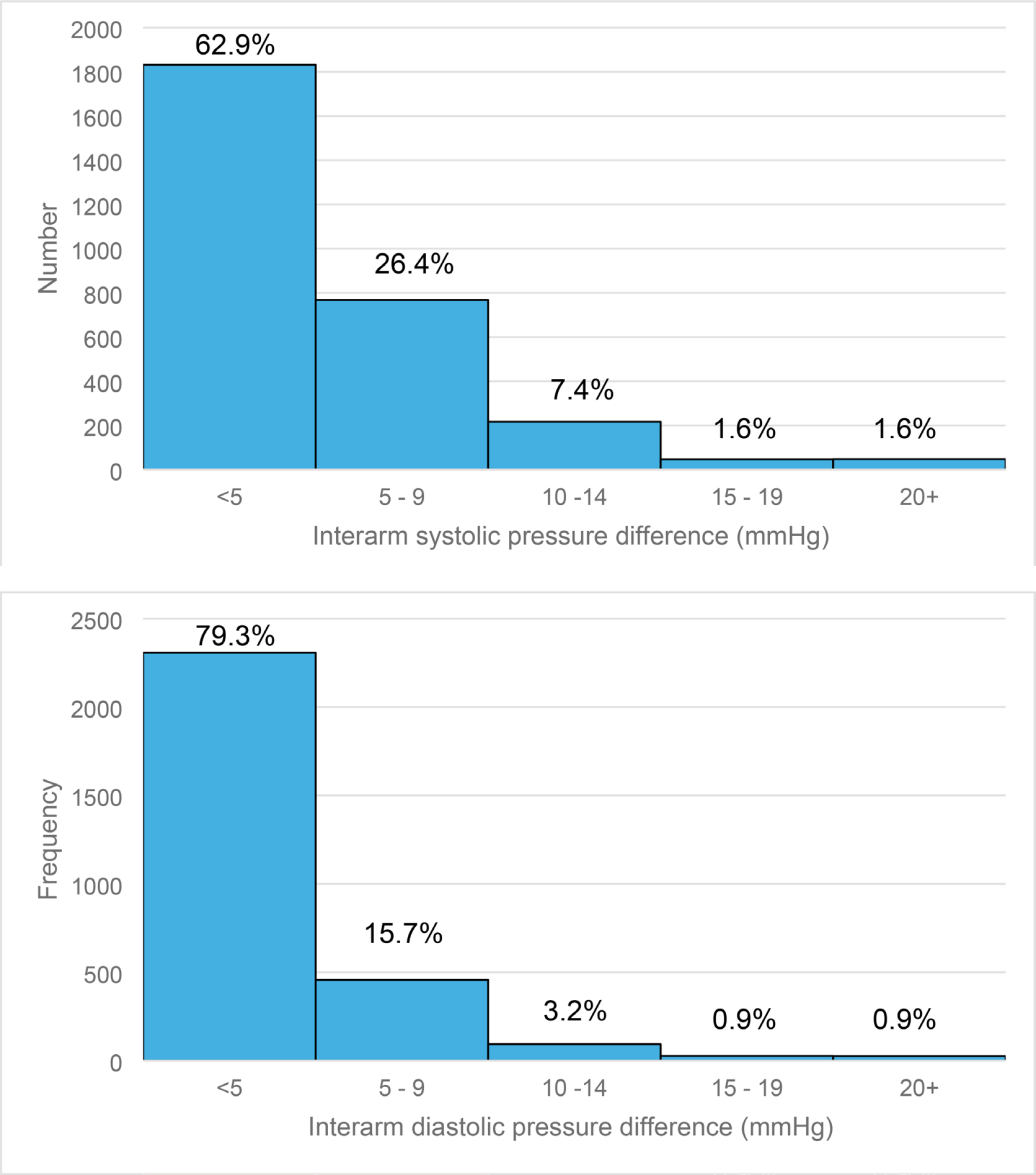
Interarm systolic and diastolic pressure difference distribution

Systolic IADs ≥5 mmHg, ≥10 mmHg, ≥15 mmHg, and ≥20 mmHg were found in 1080 (37.1%, 95% CI = 35.3 to 38.8), 312 (10.7%. 95% CI = 9.6 to 11.8), 95 (3.3%, 95% CI = 2.6 to 3.9), and 48 (1.6%, 95% CI = 1.2 to 2.1) participants, respectively. Diastolic IADs ≥5 mmHg, ≥10 mmHg, ≥15 mmHg, and ≥20 mmHg were found in 603 (20.7%, 95% CI = 19.2 to 22.2), 146 (5.0%, 95% CI = 4.2 to 5.8), 53 (1.8%, 95% CI = 1.3 to 2.3), and 26 (0.9%, 95% CI = 0.6 to 1.2) participants, respectively (Figure 1).

In 26.2% of participants higher systolic and diastolic pressure were in different arms. For these 763 participants, 8.1% had a systolic IAD of ≥10 mm Hg and 4.7% had a diastolic IAD ≥10 mm Hg.

On bivariate analysis (supplementary table 1), categorical factors associated with a systolic IAD ≥10 mmHg were hypertension, treated hypertension, never versus ever smoked, increasing BMI category, lower education level, and island site (*P* <0.05). For the continuous variables, mean BMI and systolic and diastolic pressures in the higher reading arms were higher in those with ≥10 mmHg compared with <10 mmHg systolic IAD (*P* <0.05). Except for education level the same factors were associated with a systolic IAD ≥15 mmHg. Additionally, mean ASCVD risk score was higher in those with ≥15 mmHg IAD (*P*<0.05). Supplementary table 2 shows the factors associated with IAD as a continuous variable and island site as a random effect.

On multivariable logistic and linear regression, increasing BMI along with hypertension, systolic pressure and diastolic pressure in the different models were independently associated (*P*<0.05) with systolic IADs ≥10 mmHg and ≥15 mmHg and increasing systolic IAD as a continuous variable (Tables 1 and 2 and supplementary table 3). Never versus ever smoking, higher education level, and male sex were also independent predictors in some models.

**Table 1.** Unadjusted and adjusted odds ratios for predictors of systolic interarm differences of ≥10 mmHg compared with differences <10 mmHg.^a^ Mixed effect logistic regression with island site entered as a random effect in all models

Characteristic	Unadjusted odds ratio (95% CI)	*P*-value	Adjusted odds ratio (95% CI)	*P*-value
**Model 1**				
Body mass index (kg/m^2^)	1.08 (1.06 to 1.10)	<0.001	1.07 (1.05 to 1.09)	<0.001
Education level				
Not completed high school Completed high school Some college University degree	Reference 0.86 (0.64 to 1.17) 0.69 (0.49 to 0.98) 0.61 (0.41 to 0.91)	0.3439 0.0357 0.0147	Reference 0.90 (0.66 to 1.23) 0.72 (0.51 to 1.02) 0.65 (0.43 to 0.98)	0.521 0.067 0.041
Hypertension	1.95 (1.50 to 2.54)	<0.001	1.52 (1.15 to 2.00)	0.003
**Model 2**				
Body mass index (kg/m^2^)	1.08 (1.06 to 1.10)	<0.001	1.07 (1.05 to 1.09)	<0.001
Higher systolic pressure (mmHg)	1.02 (1.02 to 1.03)	<0.001	1.02 (1.01 to 1.03)	<0.001
**Model 3**				
Body mass index (kg/m^2^)	1.08 (1.06 to 1.10)	<0.001	1.07 (1.54 to 1.04)	<0.001
Higher diastolic pressure (mmHg)	1.03 (1.02 to 1.04)	<0.001	1.03 (1.02 to 1.04)	<0.001

^a^All variables with a *P*-value of <0.2 from Supplementary Table S1 were entered. Island site was entered as a random effect while the other variables were entered as fixed effects. Hypertension (model 1), systolic (model 2), and diastolic pressure (model 3) were entered into separate models. Body mass index was entered as a continuous variable only. Backward step elimination logistic regression was performed removing variables sequentially where the *P*-value was >0.05, leaving the final model as shown.

**Table 2.** Unadjusted and adjusted odds ratios for predictors of systolic interarm differences of ≥15 mmHg compared with differences <15 mmHg^a^. Mixed effect logistic regression with island site entered as a random effect in all models

Characteristic	Unadjusted odds ratio (95% CI)	*P*-value	Adjusted odds ratio (95% CI)	*P*-value
**Model 1**				
Ever smoked	0.39 (0.17 to 0.91)	0.029	0.41 (0.17 to 0.95)	0.037
Body mass index (kg/m^2^)	1.11 (1.08 to 1.14)	<0.001	1.10 (1.07 to 1.14)	<0.001
Hypertension	2.53 (1.54 to 4.21)	<0.001	1.94 (1.16 to 3.24)	0.012
**Model 2**				
Ever smoked	0.39 (0.17 to 0.91)	0.029	0.39 (0.17 to 0.92)	0.032
Body mass Index (kg/m^2^)	1.11 (1.08 to 1.14)	<0.001	1.11 (1.07 to 1.14)	<0.001
Higher systolic pressure	1.03 (1.02 to 1.03)	<0.001	1.03 (1.02 to 1.03)	<0.001
**Model 3**				
Ever smoked	0.32 (0.17 to 0.91)	0.029	0.37 (0.16 to 0.87)	0.023
Body mass index (kg/m^2^)	1.11 (1.08 to 1.14)	<0.001	1.11 (1.08 to 1.14)	<0.001
Higher diastolic pressure (mmHg)	1.04 (1.02 to 1.06)	<0.001	1.04 (1.02 to 1.05)	<0.001

^a^All variables associated with a systolic interarm difference of ≥15 mmHg on bivariate analysis with a *P*-value of <0.2 were entered. Island site was entered as a random effect while the other variables were entered as fixed effects. BMI was entered as a continuous variable only. Hypertension (model 1), systolic (model 2), and diastolic pressure (model 3) were entered into separate models. Backward step elimination logistic regression was performed removing variables sequentially where the *P*-value was >0.05, leaving the final model as shown.

On bivariate analysis, categorical factors associated with diastolic IADs ≥10 mmHg and ≥15 mmHg, were female sex, increasing BMI category, lower education level, and island site (*P*<0.05). The continuous factors were increasing BMI, high-density lipoprotein (HDL), and diastolic pressure (*P*<0.05) (Supplementary Table S1). Additionally, hypertension, diabetes, and increasing physical activity were associated with a diastolic IAD of ≥15 mmHg (*P*<0.05).

On multivariable logistic and linear regression, increasing BMI, HDL, and diastolic pressure were independent predictors (*P*<0.05) of ≥10 mmHg and ≥15 mmHg diastolic IADs (Tables 3 and 4) and increasing IAD as a continuous variable (supplementary table 4). Hypertension and systolic pressure remained significant only in the linear regression models. Physical activity level (one logistic regression model), and increasing age, and female sex (linear regression model) were also significant (*P*<0.05).

**Table 3.** Unadjusted and adjusted odds ratios for predictors of diastolic interarm differences of ≥10 mmHg compared with differences <10 mmHg.^a^ Mixed effect logistic regression with island site entered as a random effect in all models

Characteristic	Unadjusted odds ratio (95% CI)	*P*-value	Adjusted odds ratio (95% CI)	*P*-value
**Models 1 and 2^b^**				
Body mass index (kg/m^2^)	1.07 (1.05 to 1.10)	<0.001	1.08 (1.05 to 1.11)	<0.001
HDL (mg/dl)	1.01 (1.00 to 1.03)	0.050	1.02 91.00 to 1.03)	0.008
**Model 3**				
Body mass index (kg/m^2^)	1.07 (1.05 to 1.10)	<0.001	1.07 (1.04 to 1.10)	<0.001
HDL (mg/dl)	1.01 (1.00 to 1.03)	0.050	1.02 (1.01 to 1.03)	0.007
Higher diastolic pressure (mmHg)	1.05 (1.04 to 1.06)	<0.001	1.05 (1.03 to 1.07)	<0.001

^a^All variables with a *P*-value of <0.2 from Supplementary Table S1 were entered. Island site was entered as a random effect while the other variables were entered as fixed effects. Hypertension (model 1), systolic (model 2), and diastolic pressure (model 3) were entered into separate models. Body mass index was entered as a continuous variable only. Backward step elimination logistic regression was performed removing variables sequentially where the *P*-value was >0.05, leaving the final model as shown. ^b^Neither hypertension (model 1) nor systolic hypertension (model 2) remained significant after backward elimination and were therefore eliminated from the models. DHL = high-density lipoprotein

**Table 4.** Unadjusted and adjusted odds ratios for predictors of diastolic interarm differences of ≥15 mmHg compared with differences <15 mmHg.^a^ Mixed effect logistic regression with island site entered as a random effect in all models

Characteristic	Unadjusted odds ratio (95% CI)	*P*-value	Adjusted odds ratio (95% CI)	*P*-value
Models 1 and 2^b^				
Body mass index (kg/m^2^)	1.07 (1.03 to 1.11)	0.001	1.07 (1.02 to 1.12)	0.004
HDL (mg/dl)	1.02 (1.00 to 1.04)	0.061	1.02 (1.00 to 1.04)	0.044
Physical activity				
Low Moderate High	Reference 1.66 (0.73 to 3.75) 2.02 (1.01 to 4.05)	0.223 0.046	Reference 2.35 (0.93 to 5.92) 2.39 (1.03 to 5.57)	0.071 0.046
Model 3				
Body mass index (kg/m^2^)	1.07 (1.03 to 1.11)	0.001	1.07 (1.02 to 1.12)	0.004
HDL (mg/dl)	1.02 (1.00 to 1.04)	0.061	1.02 (1.10 to 1.04)	0.043
Higher diastolic pressure	1.06 (1.04 to 1.08)	<0.001	1.06 (1.04 to 1.09)	<0.001

^a^All variables associated with a diastolic interarm difference of ≥15 mmHg on bivariate analysis with a *P-*value of <0.2 were entered. Island site was entered as a random effect while the other variables were entered as fixed effects. Body mass index was entered as a continuous variable only. Hypertension, systolic, and diastolic pressure were entered into separate models. Backward step elimination regression was performed removing variables sequentially where the *P-*value was >0.05, leaving the final model as shown. ^b^Neither hypertension (model 1) nor systolic hypertension (model 2) remained significant after backward elimination and were therefore eliminated from the models. HDL = high-density lipoprotein

### BP threshold classification using higher versus lower systolic and diastolic pressures

Higher versus lower arm systolic BP reclassified 299 (10.3%; 95% CI = 7.8 to 12.8) and 241 (8.3%; 95% CI = 5.9 to 10.7) participants at the 130-mmHg and 140-mmHg cut-offs, respectively. For diastolic BP, 316 (10.8%; 95% CI = 8.2 to 13.3) and 202 (6.9%; 95% CI = 4.9 to 8.9) participants were reclassified at the 80-mmHg and 90-mmHg cut-offs (Table 5).

**Table 5.** Comparison of proportion of participants above blood pressure (BP) and atherosclerotic cardiovascular disease (ASCVD) 10-year risk thresholds^a^ using the arms with higher and lower BP readings

		Higher arm	Lower arm	Difference % (95% CI)
	*n*	%	%	
Systolic BP ≥130 mmHg	2912	60.7 (58.9 to 62.5)	50.4 (48.6 to 52.2)	10.3 (7.8 to 12.8)
Systolic BP ≥140 mmHg	2912	38.5 (36.7 to 40.3)	30.2 (28.5 to 31.9)	8.3 (5.9 to 10.7)
Diastolic BP ≥80 mmHg	2910	55.2 (53.4 to 57)	44.4 (42.6 to 46.2)	10.8 (8.2 to 13.3)
Diastolic BP ≥90 mmHg	2910	22.2 (20.7 to 23.7)	15.3 (14 to 16.6)	6.9 (4.9 to 8.9)
10-year ASCVD risk ≥7.5%	1971	41.7 (39.5 to 43.9)	39.9 (37.8 to 42.1)	1.8 (1.2 to 2.4)
10-year ASCVD risk ≥10%	1971	32.8 (30.8 to 34.9)	30.4 (28.4 to 32.4)	2.4 (1.8 to 3.1)
10-year ASCVD risk^b^ ≥7.5%	195^b^	45.6 (38.6 to 52.6)	40.5 (33.6 to 47.4)	5.1 (-4.7 to 14.9)
10-year ASCVD risk^b^ ≥10%	195^b^	36.4 (29.6 to 43.2)	27.2 (21 to 33.4)	9.2 (0.0 to 18.4)

^a^ASCVD risk calculation according to American College of Cardiology/American Hypertension Association (ACC/AHA) guidelines.^23^ ^b^Only people with interarm BP difference ≥10 mmHg included in this comparison. BP = blood pressure

### ASCVD risk classification using higher and lower systolic pressure

Using the higher versus lower systolic pressures, 1.8% and 2.4% of participants would have their 10-year ASCVD risk reclassified from below to above the ≥7.5% and ≥10% thresholds, respectively. Of 1971 participants with an ASCVD 10-year risk score, 195 (9.9%) had a systolic IAD ≥10 mmHg. For these 195 participants, 5.1% (95% CI = -4.7 to 14.9) and 9.2% (95% CI = 0.0 to 18.4) moved from below to above the ≥7.5% and ≥10% 10-year ASCVD risk thresholds, respectively (Table 5). Only 53 people had systolic IADs ≥15 mmHg and the difference in ASCVD risk between higher and lower reading arms was not significant.

## Discussion

### Summary

The impact of measuring BP in both arms has not been previously reported in Caribbean populations. Systolic and diastolic IADs ≥10 mmHg were found in 1 in 9 and 1 in 20 participants, respectively. Only 1 in 33 and 1 in 56 had a systolic or diastolic IAD ≥15 mmHg, respectively. Increasing BMI and diastolic pressure were associated with systolic and diastolic IADs ≥10 mmHg or ≥15 mmHg. Increasing systolic pressure and hypertension were also associated with systolic IADs ≥10 mmHg or ≥15 mmHg.

Importantly, 1 in 10 and 1 in 12 would have their BP diagnosis and control status reclassified over the 130-mmHg and 140-mmHg systolic pressure thresholds, respectively, by using the higher versus the lower reading arm. For the subset with ≥10 mmHg systolic IAD, 1 in 11 would be reclassified at the 10% 10-year ASCVD risk threshold. ASCVD risk for this subset of persons has not been previously reported. Detection of a 10 mmHg IAD, and BP control and ASCVD risk reclassification all require management changes to be considered.

### Strengths and limitations

The ECHORN cohort recruited a representative sample of community-dwelling adults, making the findings relevant to primary care. Measuring BP simultaneously in both arms and repeated automatically is more accurate than sequential measurement.^8–10^

Men were under-represented. A larger sample size is needed to improve the precision of ASCVD risk estimation for those with 10 mmHg and 15 mmHg IADs.

### Comparison with existing literature

#### Interarm pressure difference prevalence and associated factors

Regarding interarm pressure difference prevalence and associated factors, BP measurement protocols and population characteristics may both contribute to an IAD. The ≥10 mmHg systolic IAD prevalence was 9.4% (mean age 61.1 years) in the Framingham Heart Study,^15^ 19% (mean age 45 years) in a Benin study,^16^ and 9.1% (mean age 62.4 years) in a Japanese study.^17^ The first two studies used different protocols for sequential BP measurement while the last one had simultaneous measurement.

A systematic review estimated the prevalence of a systolic IAD ≥10 mmHg in primary care hypertensive, diabetic, and general adult populations to be 11.2%, 7.4%, and 3.6%, respectively. Prevalence was higher in Western compared with East Asian populations^10^ In our study the prevalence was 13.0% for all people with hypertension, 13.4% in treated hypertension, 11.3% in people with diabetes, and 10.7% for the entire sample. Hypertension, increasing BMI, systolic and diastolic pressure used separately in our various models were all independent predictors of increasing systolic IAD and of systolic IADs ≥10 mmHg and ≥15 mmHg. Other studies have found increasing age,^14–16^ hypertension,^16^ increasing BMI,^15,17^ and hypercholesterolemia^15–17^ to be predictors of significant systolic IADs ≥10 mmHg. The significance of our finding that never versus current and past smoking is a predictor of increasing systolic IAD and an IAD ≥15 mmHg is uncertain and does not address cause and effect.

The islands studied have different historical and cultural backgrounds. Barbados^26^ and the US Virgin Islands^27^ with a greater proportion of their population of African descent compared with the other sites^28,29^ had a higher prevalence of IADs10 mmHg. Whether ethnicity rather than a combination of other island specific factors explains this is uncertain. The intraclass coefficients estimate the percentage of the variance owing to clustering of the sample by island and while small are in keeping with human studies.^30^

#### BP diagnosis or control reclassification

When considering BP diagnosis or control reclassification, the INTERPRESS-IPD collaboration meta-analysis estimated that 12% of participants would be reclassified at either the 130-mmHg or 140-mmHg threshold by using the higher versus lower arm systolic reading.^11^ However, included studies recruited not only community-dwelling adults but also those attending clinics for specific reasons, including vascular disease, BP was assessed mainly by sequential measurement, and in some cases by mercury sphygmomanometers and in the supine position.^12^

Data on reclassification of BP based on diastolic thresholds are sparse. In our study, despite the relative infrequency of diastolic compared with systolic IADs ≥10 mmHg, the impact on BP reclassification based on established diastolic and systolic thresholds was similar.

#### ASCVD 10-year risk reclassification

Regarding ASCVD 10-year risk reclassification, a practitioner might only use the higher reading arm for follow-up care when there is at least a 10 mmHg IAD. The INTERPRESS-IPD collaboration meta-analysis found that for all people 3.5% would be reclassified at the 10% 10-year risk threshold^11^ compared with 2.4% in our study. In our population exclusively recruited from the community and with a similar mean age, the proportions above the 10% 10-year ASCVD risk using the higher and lower systolic readings (32.8% and 30.4%, respectively) is approximately half that of the INTERPRESS-IPD collaboration (62% and 58.6%).

### Implications for practice

Classification of people from below to above guideline thresholds has consequences for treatment, follow-up, and cost of care. Intensified medication treatment may increase side effects and labelling can cause psychological distress. Evidence is lacking that measuring BP in both arms will lead to a better outcome and our study design does not answer this question.

Our study provides robust evidence that using the higher versus lower reading arm will move approximately 1 in 10 people in a diverse Caribbean population over recognised diagnosis or treatment thresholds. However, in clinical practice the effect would be half of this in cases where a physician is assessing one arm only. If an IAD of ≥15 mmHg is chosen as the threshold of normal^3^ then the number needed to screen will be high. Practitioners wanting to implement guidelines may face barriers. Measuring BP in both arms takes time and is not always reproducible. With sequential readings, the first reading is often higher than the second^8,31^ and higher systolic and diastolic readings occurring in different arms may cause uncertainty. Practitioners would need devices capable of measuring BP in both arms simultaneously but information on the extent to which IADs are sustained over time comes mainly from studies that have repeated assessments at one visit or after a few months.^12,32^ Wave 2 of the ECHORN project will provide longitudinal data on our study participants thus allowing a determination of whether the IADs we reported are sustained over a 4–5-year period. It may also provide guidance as to the frequency at which IADs need to be reassessed after initial assessment.

## Data

The dataset relied on in this article is available from the corresponding author on reasonable request.
